# Effect of remifentanil-based fast-track anesthesia on postoperative analgesia and sedation in adult patients undergoing transthoracic device closure of ventricular septal defect

**DOI:** 10.1186/s13019-020-01339-0

**Published:** 2020-09-29

**Authors:** Ning Xu, Shu-Ting Huang, Kai-Peng Sun, Liang-Wan Chen, Qiang Chen, Hua Cao

**Affiliations:** 1 Department of Cardiac Surgery, Fujian Maternity and Child Health Hospital, Affiliated Hospital of Fujian Medical University, Fuzhou, China; 2 Department of Cardiovascular Surgery, Union Hospital, Fujian Medical University, Fuzhou, China

**Keywords:** Remifentanil, Fast-track anesthesia, Device, VSD

## Abstract

**Objective:**

To investigate the effect of remifentanil-based fast-track anesthesia on analgesia and sedation after transthoracic device closure of ventricular septal defects (VSDs) in adult patients.

**Methods:**

A retrospective analysis was performed on 59 patients aged 21–53 years who underwent transthoracic device closure of VSDs from January 2019 to September 2019. According to the different anesthesia strategies, the patients were divided into the R group (using remifentanil-based anesthesia, *n* = 33) and the S group (using sufentanil-based anesthesia, *n* = 26). Patient-related clinical data, postoperative analgesia, and sedation scores were collected and analyzed.

**Results:**

There was no significant difference in age, gender, body weight, and operation time between the group R and the group S (*P* > 0.05). There was also no significant difference in intraoperative hemodynamic changes, BIS scores, postoperative analgesia, and sedation scores between the two groups (*P* > 0.05). The duration of mechanical ventilation, the length of ICU stay, and hospital stay in the group R were significantly lower than those in the group S (*P* < 0.05).

**Conclusion:**

Remifentanil-based fast-track anesthesia is effective for adult patients undergoing transthoracic device closure of VSDs, which may shorten the mechanical ventilation duration, the ICU and hospital stay of patients.

## Introduction

Ventricular septal defect (VSD) is one of the most common congenital heart diseases requiring surgical correction. In Asia, it accounts for 30% of all patients with congenital heart disease [1]. The main treatment methods are traditional surgical repair, percutaneous device closure, and transthoracic device closure. Although traditional surgical repair has a wide range of indications, it is combined with the following deficiencies: large incision and trauma, low aesthetic effect, long operation time and hospital stay, high risk of postoperative complications, etc. Percutaneous device closure has the advantage of no incision and fast recovery but has the disadvantage of X-ray exposure. In recent years, our center has tried to treat the simple VSD using transthoracic device closure and has achieved good results [2–4]. In order to meet the needs of short anesthesia time and early tracheal extubation after transthoracic device closure of VSDs, fast-track anesthesia was adopted in our cardiac center. Fast-track anesthesia has been applied and studied in adult congenital heart disease surgery in many countries. This study aimed to investigate the effect of remifentanil-based fast-track anesthesia on analgesia and sedation after transthoracic device closure of VSDs under transesophageal guidance echocardiography (TEE) in adult patients.

## Materials and methods

A retrospective analysis was conducted on 59 patients who underwent transthoracic device closure of VSDs from January 2019 to September 2019. All patients were diagnosed as simple perimembranous VSD with or without mild pulmonary hypertension, no history of cardiovascular surgery, and other systemic diseases, no history of opioid drug allergy. All patients or their close relatives signed the informed consent forms. All patients were routinely examined preoperatively after admission and were determined to have no contraindications to anesthesia and surgery. Then all patients were informed of the different anesthesia strategies and related risks before surgery. After considering the patient’s physical condition and the patient’s wishes, the anesthesiologist and surgeon determined the anesthesia strategy of the patient and divided the patients into the group R (using remifentanil-based anesthesia, *n* = 33) and the group S (using sufentanil-based anesthesia, *n* = 26) according to different anesthesia strategies. A team of identical surgeons and anesthesiologists worked together to perform all the operations.

In the group R, all patients fasted of food for 6 to 8 h and water for 2 to 3 h. After entering the operating room, the electrocardiogram and the saturation of peripheral oxygen (SpO2) were monitored. After peripheral venous access was established, a continuous drip of normal saline was given. During anesthesia induction, remifentanil was intravenously injected at 1.5 μg/kg, and atracurium cisbenzoate was injected at 0.5 mg/kg. After muscle relaxation, tracheal intubation and mechanical ventilation were performed. The ventilation mode was a pressure control mode, and the end-expiratory carbon dioxide partial pressure was monitored. Radial artery puncture was performed to monitor arterial blood pressure directly, and subclavian vein puncture was performed to monitor central venous pressure. Anesthesia was maintained with 2–3% sevoflurane and 40% oxygen inhalation and a continuous remifentanil-based infusion at the rate of 0.2–0.5 μg/kg/min intravenous pump. The dose was adjusted according to anesthesia’s depth during the operation to maintain the BIS value at 40–60. The operating room temperature was maintained at about 25 °C, and the infusion was not too cold or too quick. The infusion was heated appropriately to maintain the patient’s body temperature above 36.5 °C.

In the group S, preoperative treatment was the same as that in the group R. During the induction of anesthesia, sufentanil was intravenously injected at 1 μg/kg, and atracurium cisbenzoate was injected at 0.5 mg/kg. Anesthesia was maintained with 2–3% sevoflurane and 40% oxygen inhalation and a continuous sufentanil infusion at the rate of 0.25–0.5μg/kg/h using an intravenous pump. The dose was adjusted according to anesthesia’s depth during the operation to maintain the BIS value at 40–60. The intraoperative monitoring measures were the same as those in the group R.

After general anesthesia, the patients were placed in the supine position. A small incision of approximately 3–4 cm at the lower end of the sternum was performed to access the pericardial cavity. Then, the pericardium was opened to expose the right ventricle, and heparinization (1 mg/kg) was administered intravenously. Next, TEE was used to confirm the diagnosis of a VSD, selected the appropriate size of the occluder, and determined the puncture site. A purse-string suture was performed at the right ventricle’s surface, and a needle was punctured in this site. A guidewire was inserted into the needle, then the right ventricle - VSD - left ventricle transport track was established by TEE guidance. The occluder was sent and released to close the VSD through a deliver sheath. Then, TEE was used to confirm the occluder’s position and that there was no significant residual shunt or significant aortic regurgitation. Electrocardiography was used to indicate whether a conduction block occurred. After closing the sternum, 0.2% ropivacaine was applied to the incision and sternal membrane for local infiltration anesthesia. All patients were transferred to the intensive care unit (ICU) for further monitoring and treatment.

Postoperative analgesia and sedation management was the same in both groups. The method of patient-controlled analgesia was used to perform intravenous analgesia for all patients. A standard protocol of 2.0 μg/kg sufentanil, 2.0 μg/kg dexmedetomidine, 0.2 mg/kg tropisetron and 100 ml physiological saline was used with the speed of 2 ml/h for the first postoperative 48 h. It was up to an identical team of ICU physicians and nurses to adjust the dosage, or stop the medication accordingly to the patients’ condition. Other postoperative medications and care were the same in both groups. The ICU physicians and nurses did not know the patient’s intraoperative anesthesia strategy.

The related clinical data were recorded, including age, gender, body weight, the diameter of the VSD, the occluder’s size, operation time, mechanical ventilation duration, length of ICU and hospital stay, and anesthesia-related complications. The central venous pressure, mean arterial pressure, heart rate, oxygen saturation, and BIS value were also monitored and recorded. After tracheal intubation was removed, the professional staff determined the patients’ sedative and analgesic scores at four consecutive time points (1, after extubation, 2, 30 min after extubation, 3, 60 min after extubation, and 4, before leaving the ICU).

The Richmond Agitation and Sedation Score (RASS) was used in this study, which was a 10-point scale that provided criteria for levels of sedation and agitation, as follows: + 4: Combative, + 3: Very agitated, + 2: Agitated, + 1: Restless, 0: Alert and calm, − 1: Drowsy, − 2: Light sedation, − 3: Moderate sedation, − 4: Deep sedation, − 5: Unarousable. RASS − 2 to 1 were used to defined as light sedation [5–7].

The Critical Care Pain Observation Tool (CPOT) was used to measure analgesia inpatient objectively. Four behavioral domains of pain were assessed: patient facial expression, body movements, vocalization, and muscle tension. Individual domain items were scored from 0 (no pain) to 2 (highest score for pain); total scores range from 0 to 8. A score ≧2 indicates pain [8–10].

The Numerical Rating Scale (NRS) was used to measure analgesia inpatient subjectively. An NRS allowed a person to describe the intensity of his/her pain as a number usually ranging from 0 to 10, where “0” meant “no pain” and “10” meant pain as “bad as it could be,” and the patient circled a number to represent the degree of pain. We regarded 0 as painless, 1 to 3 as mild pain, 4 to 6 as moderate pain, and 7 to 10 as severe pain [11, 12].

The data of this study were analyzed by SPSS 22.0. The continuous data (including age, weight, the diameter of the VSD, the size of the occluder, operation time, the duration of mechanical ventilation, the length of ICU and hospital stay, ejection fraction, CVP, MAP, HR, RASS score, CPOT score, NRS score, BIS value) were expressed as the mean ± standard deviation, and the incidences were represented as %. The continuous data in Table 1 were confirmed following the normal distribution, and the t-test was used. A Chi-square test was used to compare the qualitative data between the two groups. Tables 2 and 3 used repeated measure ANOVA, and the LSD method was used for pair-wise comparison between different times. *P* value < 0.05 was defined as statistically significant.

**Table 1:** Comparison of clinical data in both groups

Items	Group R	Group S	***P***-value
**Number of patients**	33	26	
**Gender (male/female)**	18/15	14/12	0.957
**Age (years)**	38.33 ± 8.49	35.62 ± 8.31	0.223
**Weight (kg)**	54.76 ± 6.63	53.69 ± 5.73	0.477
**VSD size (mm)**	5.33 ± 1.05	5.19 ± 0.94	0.594
**Occluder size (mm)**	6.39 ± 1.12	6.46 ± 1.17	0.822
**Ejection fraction (%)**	61.42 ± 5.53	64.35 ± 7.01	0.709
**Operation time (min)**	35.18 ± 5.84	34.81 ± 7.34	0.696
**Mechanical ventilation time (h)**	2.55 ± 1.15	3.35 ± 1.23	0.031
**Intensive care time (h)**	5.79 ± 1.34	10.73 ± 2.54	0.047
**Hospital stay (days)**	2.12 ± 0.74	4.38 ± 1.36	0.041

*Abbreviations*: *VSD* Ventricular septal defect

**Table 2:** Comparison of interoperative hemodynamic data in both groups during the procedure

Items	Group R	Group S	***P***-value
**After intubation**			
**CVP (mmH2O)**	7.42 ± 1.03	6.97 ± 1.24	0.574
**MAP (mmHg)**	95.68 ± 9.77	94.34 ± 8.63	0.681
**HR (beats/min)**	118.91 ± 15.33	116.37 ± 14.69	0.826
**BIS value**	52.78 ± 8.28	54.37 ± 8.63	0.782
**Skin incision**			
**CVP (mmH2O)**	7.96 ± 1.42	7.35 ± 1.39	0.698
**MAP (mmHg)**	98.32 ± 8.97	101.04 ± 9.73	0.743
**HR (beats/min)**	119.63 ± 18.76	116.89 ± 15.78	0.752
**BIS value**	44.52 ± 6.73	46.32 ± 7.41	0.748
**Occluder release**			
**CVP (mmH2O)**	8.13 ± 2.03	7.04 ± 1.96	0.728
**MAP (mmHg)**	96.77 ± 8.95	98.23 ± 9.16	0.747
**HR (beats/min)**	124.32 ± 17.41	126.37 ± 18.44	0.821
**BIS value**	46.39 ± 6.42	47.75 ± 6.89	0.882
**After procedure**			
**CVP (mmH2O)**	7.42 ± 1.75	7.82 ± 1.59	0.673
**MAP (mmHg)**	98.64 ± 8.75	97.36 ± 9.21	0.796
**HR (beats/min)**	122.85 ± 20.96	119.88 ± 21.83	0.746
**BIS value**	48.38 ± 7.29	49.52 ± 8.09	0.723

*Abbreviations*: *CVP* Central venous pressure, *MAP* Mean arterial pressure, *HR* Heart rate

**Table 3:** Comparison of postoperative hemodynamic data in both groups

Items	Group R	Group S	***P***-value
**After entering ICU**			
**CVP (mmH**_**2**_**O)**	8.43 ± 1.93	8.52 ± 2.21	0.684
**MAP (mmHg)**	95.46 ± 9.73	96.72 ± 8.59	0.771
**HR (beats/min)**	124.37 ± 18.92	126.58 ± 19.66	0.684
**RASS score**	−1.27 ± 0.68	−0.98 ± 0.74	0.785
**CPOT score**	1.25 ± 1.09	1.37 ± 1.20	0.881
**NRS score**	0.58 ± 0.66	0.73 ± 0.67	0.731
**After extubation**			
**CVP (mmH**_**2**_**O)**	8.64 ± 2.03	9.03 ± 2.31	0.665
**MAP (mmHg)**	98.32 ± 9.65	97.68 ± 8.47	0.743
**HR (beats/min)**	125.85 ± 18.85	127.44 ± 17.95	0.721
**RASS score**	−0.43 ± 0.82	−0.29 ± 0.92	0.649
**CPOT score**	1.53 ± 1.28	1.61 ± 1.31	0.820
**NRS score**	2.48 ± 1.03	2.77 ± 1.25	0.742
**After entering ICU 30 mins**			
**CVP (mmH**_**2**_**O)**	9.13 ± 2.26	8.93 ± 2.17	0.762
**MAP (mmHg)**	97.66 ± 8.52	98.74 ± 8.94	0.787
**HR (beats/min)**	124.76 ± 18.96	126.30 ± 19.27	0.683
**RASS score**	−0.55 ± 0.85	−0.35 ± 0.74	0.732
**CPOT score**	2.73 ± 1.04	3.11 ± 1.07	0.615
**NRS score**	2.85 ± 1.15	2.96 ± 1.21	0.694
**Before leaving ICU**			
**CVP (mmH**_**2**_**O)**	9.22 ± 2.15	9.17 ± 2.24	0.638
**MAP (mmHg)**	96.57 ± 8.94	98.27 ± 9.66	0.686
**HR (beats/min)**	125.83 ± 19.75	127.86 ± 18.35	0.773
**RASS score**	−0.34 ± 0.63	−0.38 ± 0.71	0.783
**CPOT score**	2.84 ± 1.17	3.26 ± 1.09	0.669
**NRS score**	3.05 ± 1.35	3.10 ± 1.29	0.638

## Results

There was no significant difference in age (*p* = 0.223), gender (*p* = 0.957), body weight (*p* = 0.477), the diameter of the VSD (*p* = 0.594), the size of the occluder (*p* = 0.822), ejection fraction (*p* = 0.709) and operation time (*p* = 0.696) between the two groups. Table 1 showed that the duration of mechanical ventilation (*p* = 0.031), the length of ICU stay (*p* = 0.047), and the length of hospital stay (*p* = 0.041) in the group R were significantly shorter than those in the group S. Table 2 showed that there were no significant differences in the changes in intraoperative hemodynamic data. Table 3 showed the relevant clinical data at four consecutive time points. The central venous pressure, mean arterial pressure, and heart rate of the two groups were in the normal range during the whole monitoring process, and there was no significant difference between the two groups at each time point (*P* > 0.05). Moreover, there was no significant difference between the two groups in the RASS score, the CPOT score, and the NRS scale score (*P* > 0.05). There were no anesthesia-related complications and side effects associated with narcotic drugs in the two groups.

## Discussion

VSD is one of the most common congenital heart diseases requiring correction, and the perimembranous VSD is the most common one, accounting for about 60% ~ 70% [1]. The primary methods for the treatment of VSD are as follows: traditional surgical repair under cardiopulmonary bypass, percutaneous device closure, and transthoracic device closure. Except for the traditional surgical approach, all other device closure approaches have their limitations in indications. The standard surgical method for adult patients with VSD has the characteristics of a large incision, internal steel wire, noticeable surgical scar. The puncture of a femoral vein performs percutaneous device closure without a significant surgical incision. However, the percutaneous approach can be challenging because the patient has to undergo multiple exposures to X-ray radiation, which is particularly inappropriate for pregnant women and children [13, 14]. The location and the surrounding structure of the VSD are essential factors in the procedure success. Many kinds of literature have shown that the transthoracic device closure of VSD not only virtually ensures the therapeutic effect, but also reduces the length of the incision, reduces the postoperative pain, and shortens the length of hospital stay [3, 4].

To meet the needs of early extubation and short ICU stay after transthoracic device closure of VSD, fast-track anesthesia technology was used in our center. The fast-track anesthesia technique, which means to help patients regain consciousness and spontaneous breathing as soon as possible, and usually tracheal extubation within 6 h after operation [15–18]. Fast-track cardiac anesthesia technology is not only an anesthetic measure to help patients recover quickly after the operation but also one of the improvement measures to accelerate the anesthesia management in the perioperative period of rehabilitation.

Fast-track cardiac anesthesia has been used in congenital cardiac surgery at home and abroad, but its application in transthoracic device closure of VSD in adult patients under TEE’s guidance was rarely reported [19]. In this study, we did such research to find more reasonable anesthetics and anesthetic strategies to achieve the goal of early postoperative tracheal extubation, rapid recovery and discharge of patients, saving medical resources, and reducing patients’ burden. In recent years, many randomized controlled trials and meta-analysis showed that fast-track anesthesia led to patients’ relatively low risk compared with conventional anesthesia [20]. A systematic review meta-analysis by Alghamdi et al. showed that both fast-track and conventional anesthesia methods were safe and effective in terms of mortality and the incidence of severe postoperative complications, but fast-track anesthesia management could shorten the intubation time and the length of ICU stay of patients after operation [21–23].

Long-term endotracheal intubation and prolonged mechanical ventilation were the main risk factors for increased postoperative respiratory-related complications [24]. At present, a large number of studies had shown that compared with conventional anesthesia management in cardiac surgery, tracheal extubation in the operating room after the operation could reduce the use of muscle relaxant drugs for patients, enabled them to resume spontaneous breathing as soon as possible, and reduced the risk of ventilator related iatrogenic pneumonia and damage to respiratory tract caused by long-term catheterization, and other pulmonary complications [25–27]. A logistic regression analysis study based on propensity matching score showed that the mode shift from conventional anesthesia management to fast-track anesthesia management could improve cost-effectiveness in low- and medium-risk patients undergoing cardiac surgery [28].

The results of this study showed that the dosage of anesthetics in the group R was lower, indicating that remifentanil-based fast-track anesthesia could reduce the dose of anesthetics in adult patients with VSD. This was closely related to the induction of sevoflurane in fast-track anesthesia, reduced long-acting opioids, and reduced narcotic analgesics’ dosage. At the same time, this study also found no significant difference in intraoperative hemodynamic monitoring indexes between the two groups, which indicated that remifentanil-based fast-track anesthesia had no adverse effect on the surgical process adult patients with VSD. Remifentanil-based fast-track anesthesia was not only beneficial for extubation as soon as possible after operation, but also could play a similar anesthetic effect with sufentanil-based anesthesia. The duration of postoperative mechanical ventilation, the length of ICU stay, and hospital stay in the group R was significantly lower than those in the group S, which indicated that remifentanil-based fast-track anesthesia had apparent advantages in these aspects. As a short-acting opioid, remifentanil was used for fast-track anesthesia, with good anesthetic effect and short duration. Sevoflurane was a commonly used inhalation anesthetic, and atracurium was an auxiliary anesthetic; all of them were safe and effective and high tolerance for the patients [29]. Fast-track anesthesia could effectively avoid stress stimulation of patients, promote the prognosis of patients, improve the effectiveness of surgical treatment, and reduce the days of hospitalization [30].

Sedation and analgesia are the actual contents of perioperative and postoperative anesthesia management. To improve the clinical effect and achieve early extubation, reducing the use of long-lasting opioids had become one of the hotspots of clinical research. Patients with an unreadable pain expression, resulting in excessive intravenous analgesic medication, should be prone to adverse reactions. In the results of this study, there was no significant difference in postoperative RASS sedation scores between the two groups. The CPOT scores and NRS scores of the two groups were in the range of mild pain, and there was also no significant difference between the two groups. During the monitoring time, the patients’ vital signs were stable, and there was no severe delirium of agitation and drowsiness, which was consistent with the results of foreign studies. Besides, there were no anesthesia-related complications such as nausea, vomiting, dizziness, headache, respiratory depression, etc. The results also confirmed that remifentanil-based fast-track anesthesia was effective in transthoracic device closure of VSD in adult patients, and it could shorten the duration of mechanical ventilation, reduce the length of ICU and hospital stay.

### Limitation

There were still some limitations to this study. This study was a retrospective single-center study that involved a small sample size and might have some selective deviation in case enrollment. It was not possible to be double-blind in the choice of anesthesia strategies, and it might have a significant effect on the results, but we still thought that our paper had some clinical significance. Therefore, the conclusion might not be extended to the cardiac operation under cardiopulmonary bypass and low patients’ general conditions. Besides, due to limited conditions, this paper failed to comprehensively collect the data comparison of complications of fast-track anesthesia and other conventional anesthesia strategies. Although the existing article confirmed the fast-track anesthesia’s safety, the research on security in this study was still insufficient. A more extensive and multicenter study needs to be done to determine whether our conclusions were correct and feasible.

## Conclusion

Remifentanil-based fast-track could be effectively used in adult patients who underwent transthoracic device closure of VSDs, which could shorten mechanical ventilation duration, reduce the length of ICU and hospital stay, and show adequate postoperative analgesia and sedation effect.

## Data Availability

Data sharing not applicable to this article as no data sets were generated or analyzed during the current study.

## Abbreviations

VSD: Ventricular septal defects
TEE: Transesophageal echocardiography
ICU: Intensive care unit
RASS: Richmond Agitation and Sedation Score
CPOT: Critical Care Pain Observation Tool
NRS: Numerical Rating Scale
